# Genome sequence and analysis of a Japanese radish (*Raphanus sativus*) cultivar named ‘Sakurajima Daikon’ possessing giant root

**DOI:** 10.1093/dnares/dsaa010

**Published:** 2020-05-19

**Authors:** Kenta Shirasawa, Hideki Hirakawa, Nobuko Fukino, Hiroyasu Kitashiba, Sachiko Isobe

**Affiliations:** 1 Kazusa DNA Research Institute, Kisarazu, Chiba 292-0818, Japan; 2 Institute of Vegetable and Floriculture Science, NARO, Tsu, Mie 514-2392, Japan; 3 Graduate School of Agricultural Science, Tohoku University, Sendai, Miyagi 980-0845, Japan

**Keywords:** radish, chromosome-scale pseudomolecule sequences, long-read sequence technology, genome sequence

## Abstract

**Aim:**

The complex genome of a Japanese radish (*Raphanus sativus*) cultivar named ‘Okute-Sakurajima’ with an extremely large edible round root was analysed to explore its genomic characteristics.

**Methods and Results:**

Single-molecule real-time technology was used to obtain long sequence reads to cover 60× of the genome. *De novo* assembly generated 504.5 Mb contigs consisting of 1,437 sequences with the N50 value of 1.2 Mb and included 94.1% of the core eukaryotic genes. Nine pseudomolecules, comprising 69.3% of the assembled contigs, were generated along with a high-density SNP genetic map. The sequence data thus established revealed the presence of structural variations and rearrangements in the *Brassicaceae* genomes.

**Conclusion and perspective:**

A total of 89,915 genes were identified in the ‘Okute-Sakurajima’ genome, 30,033 of which were newly found in this study. The genome information reported here will not only contribute to the establishment of a new resource for the radish genomics but also provide insights into the molecular mechanisms underlying formation of the giant root.

## 1. Introduction

Daikon, or Japanese radish (*Raphanus sativus*), belongs to *Brassicaceae* with its root varying considerably in size and shape among different cultivars.^1^ ‘Sakurajima daikon’ is the name given to a group of the radish varieties developed and cultivated mainly in Kagoshima prefecture of Japan. The soil in the region of its cultivation contains volcanic ashes originated from Mt. Sakurajima which are said to be particularly suited for cultivating ‘Sakurajima daikon’. One well-known ‘Sakurajima daikon’ cultivar is named ‘Okute-Sakurajima’, which has a large round root often exceeding 20 kg in weight.^1^ The size and shape of ‘Okute-Sakurajima’ root are attractive traits in plant breeding, but the molecular mechanisms underpinning these characteristics remain largely unestablished. 

Four genome assemblies based on next-generation sequencing technologies have been reported for radish,^2–5^ but they do not include ‘Sakurajima daikon’, in particular ‘Okute-Sakurajima’. The sequence contiguities of the genome assemblies in these reports are relatively short and do not cover the entire radish genome.^6^ One reason for the short sequences in the assembly may be due to the complexity of the radish genome, because species belonging to *Raphanus*, like those in the genus *Brassica*, underwent genome triplication at a certain point after divergence from an ancestor common to *Arabidopsis*.^7^ Furthermore, together with other *Brassica* species, radish is self-incompatible and allogamous, resulting in a highly heterozygous genome.^8^

Recent advances in long-read sequence technology have enabled us to obtain the genomic sequence data of highly heterozygous plant species.^9^ In this study, we attempted to sequence the ‘Okute-Sakurajima’ genome using the long-read sequencing technology and the sequences thus obtained were aligned to a high-density SNP genetic map to produce chromosome-scale pseudomolecule sequences. This has substantially improved the previous radish genome assemblies that contained gaps. The resultant radish genome data of better quality will provide clues as to its evolutionary history, in particular with regard to the molecular events that resulted in giant root formation.

## 2. Materials and methods

### *2.1. De novo* assembly of the ‘Okute-Sakurajima’ genome

Total DNA was extracted from young leaves of the ‘Okute-Sakurajima’ radish cultivar (NARO GeneBank accession number: JP27228) using a Genomic-tip kit of Qiagen (Hilden, Germany). Short-read sequence data were obtained using a MIGSEQ-2000 DNA sequencer (also known as a DNBSEQ-G400; MGI Tech, Shenzhen, China) and were used to estimate the genome size after removing adaptor sequences (AAGTCGGAGGCCAAGCGGTCTTAGGAAGACAA and AAGTCGGATCGTAGCCATGTCGTTCTGTGAGCCAAGGAGTTG) and reads from organelle genomes (GenBank accession numbers: NC_018551 and NC_024469). Subsequently, *k*-mer distribution analysis was performed using Jellyfish. To obtain long-read sequence data, an SMRT sequence library was constructed with an SMRTbell Express Template Prep Kit (PacBio, Menlo Park, CA, USA) and used to sequence on a PacBio Sequel system (PacBio). The long-read sequences thus obtained were assembled, and the two haplotype sequences of the diploid genome were resolved with Falcon-unzip. Errors in the resultant assembled data were corrected twice with arrow to obtain the final assembly designated as RSAskr_r1.0. The completeness evaluation of the assembly was performed with BUSCO (embryophyta odb9). The software tools used for genome assembly are shown in Supplementary Table S1.

### 2.2. Construction of pseudomolecules based on the genetic map

An F_2_ mapping population (*n* = 115), termed SNF2, was derived from a cross between an inbred line via self-pollination of radish cultivar ‘Shogoin Daikon’ and a line of *R. sativus* var. *raphanistroides* ‘Nohara 1’ which was collected from Nohara, Maizuru, Kyoto, Japan, and was used to establish genetic maps according to the methods of Shirasawa and Kitashiba.^6^ In brief, DNA was extracted from leaves of each line and used for ddRAD-Seq library construction. The library was sequenced on a HiSeq4000 sequencer (Illumina, San Diego, CA, USA) and the data obtained were analysed as described by Shirasawa and Kitashiba.^6^ After discarding low-quality sequences and adapter sequences using FASTX-Toolkit and PRINSEQ, the resultant high-quality reads were mapped onto the RSAskr_r1.0 assembly, using Bowtie2 to call SNPs using the mpileup command in SAMtools followed by filtering out the low-quality data with VCFtools. In parallel, ddRAD-Seq data (DRA accession number: DRA005069) from another F_2_ population (*n* = 95) named ASF2,^2^ which was derived from a cross between ‘Aokubi *S-h*’ and ‘Sayatori 26704’, were also analysed as described above. SNP data were used for genetic map construction with Lep-Map3. The RSAskr_r1.0 sequence assembly was assigned to the genetic maps with ALLMAPS to establish pseudomolecule sequences representing the radish chromosome-scale sequences, termed RSAskr_r1.0.pmol. The structure of the ‘Okute-Sakurajima’ genome was compared with those of *R. sativus*,^4^*Brassica rapa*,^10^ and *Arabidopsis thaliana*^11^ using D-Genies.

### 2.3. Identification of genes and repeats in the genome sequences

*Ab initio* gene prediction was performed with the RSAskr_r1.0.pmol sequences using Augustus and intrinsic (with start and stop codons) and partial (without start and/or stop codons) genes were selected to exclude transposable elements, pseudogenes (with in-frame stop codons), and short genes (encoding <50 amino acids) as described by Kitashiba et al.^2^ The genes predicted in the RSA_r1.0 assembly^2^ and *de novo* transcriptome assembled from 73.7 Gb public RNA-Seq reads (Sequence Read Archive accession numbers: SRA072125, SRA080301, SRA140538, SRA306323, SRA401451, and SRA498899) with Trinity were mapped onto the RSAskr_r1.0.pmol pseudomolecules using Minimap2. Repetitive sequences in the RSAskr_r1.0.pmol were identified with RepeatMasker, for which repeat sequences registered in Repbase and a *de novo* repeat library built with RepeatScout, were used. The repeat elements were classed in nine types; short interspersed nuclear elements (SINEs), long interspersed nuclear elements (LINEs), long terminal repeats (LTR) elements, DNA elements, unclassifed, satellintes, simple repeats, and low complexity in accordance with RepeatMasker.

## 3. Results and data description

### *3.1. De novo* assembly of the ‘Okute-Sakurajima’ genome

**Figure 1:**
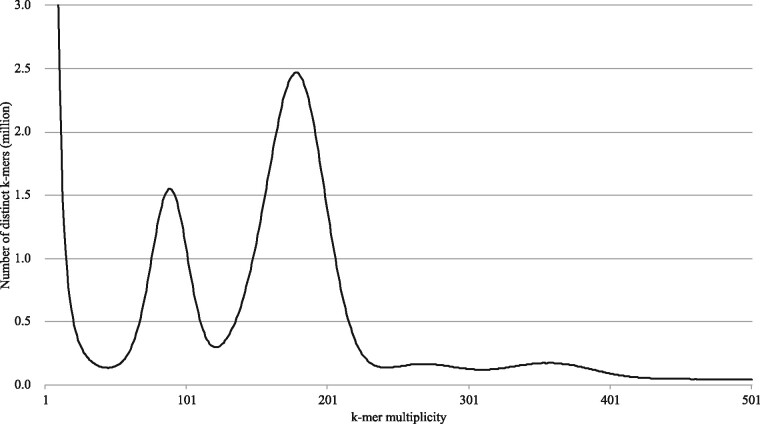
Genome size estimation for ‘Okute-Sakurajima’ with the distribution of the number of distinct *k*-mers (*k *=* *17) with the given multiplicity values.

*K*-mer distribution analysis of the 99.3-Gb high-quality short-read data indicated that the ‘Okute-Sakurajima’ genome was highly heterozygous, and that the estimated haploid genome size was 498.5 Mb (Fig. 1). Subsequent long-read sequencing produced 36.0 Gb data (60.7× coverage of the estimated genome size) in 2.3 million reads with N50 length of 29.1 kb. After two rounds of data polishing, the long-read assembly consisting of 504.5 Mb primary contigs (including 1,437 sequences with N50 length of 1.2 Mb) and 263.5 Mb alternative contigs comprised of the other haplotypes with different alleles, also known as haplotig sequences (including 2,373 sequences with N50 length of 154.6 kb) (Table 1) were obtained. A BUSCO analysis of the primary contigs indicated that 71.0% and 23.1% of sequences were single-copy complete BUSCOs and duplicated complete BUSCOs, respectively (Table 1), suggesting that most of the genes were present in the primary contigs.

**Table 1:** Statistics of the primary contig sequences of ‘Okute-Sakurajima’

	RSAskr_r1.0
Total contig size (bases)	504,534,164
Number of contigs	1,437
Contig N50 length (bases)	1,247,688
Longest contig size (bases)	8,317,732
Gap (%)	0.0
Complete BUSCOs	94.1
Single-copy BUSCOs	71.0
Duplicated BUSCOs	23.1
Fragmented BUSCOs	2.0
Missing BUSCOs	3.9
#Genes (*ab initio*)	89,915
#Genes (mapping)	78,645

### 3.2. Construction of pseudomolecules based on the genetic map

**Figure 2:**
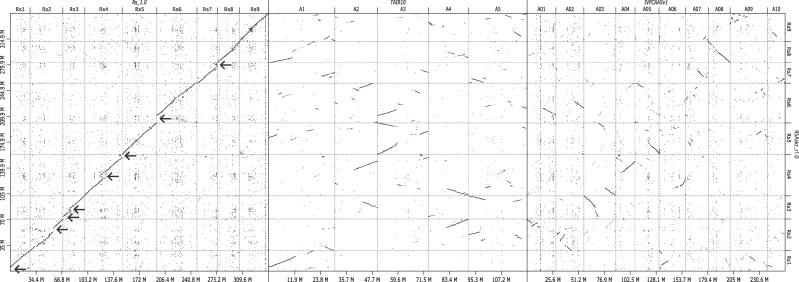
Sequence similarity of the ‘Okute-Sakurajima’ genome with three other species. Dots indicate sequence similarity of the ‘Okute-Sakurajima’ genome (RSAskr_r1.0) on the vertical axis versus those of *R. sativus* (Rs_1.0), *A. thaliana* (TAIR10), and *B. rapa* (IVFCAASv1) on the horizontal axis. Arrows indicate major regions on Rs1 to Rs7 presented in only RSAskr_r1.0.pmol.

From the sequence data thus obtained, we were able to identify 5,872 and 2,830, respectively, of high-quality SNPs in the SNF2 and ASF2 mapping populations and used them for linkage analysis. The resultant genetic map for SNF2 consisted of nine linkage groups with 5,570 SNPs covering 867.2 cM, and the map for ASF2 comprised nine linkage groups with 2,680 SNPs covering 895.3 cM (Table 2). The contig sequences of RSAskr_r1.0 were assigned to the radish chromosomes in accordance with the two genetic maps mentioned above. Sequences of the nine pseudomolecules, termed RSAskr_r1.0.pmol, were found to span 349.8 Mb (69.3%) of 293 contigs (Table 3), of which 95 sequences (218.5 Mb, 43.3%) were properly oriented. The resultant nine sequences were named Rs1 through Rs9 according to the nomenclature proposed by Shirasawa and Kitashiba.^6^ The remaining unassigned sequences (*n* = 1,144, 154.7 Mb, 30.7%) were tentatively grouped into Rs0. The structure of the ‘Okute-Sakurajima’ genome thus established was found to be conserved within *R. sativus*, but partially deviated from the *B. rapa* and *A. thaliana* genomes as indicated previously^2^ (Fig. 2). Eight major regions on Rs1 to Rs7 were present in only RSAskr_r1.0.pmol.

**Table 2:** Genetic map length and number of SNPs for F_2_ radish populations

LG^a^	SNF2^b^		ASF2^c^	
	Number of SNPs	Map length (cM)	Number of SNPs	Map length (cM)
Rs1	608	64.0	253	87.4
Rs2	757	104.9	299	103.4
Rs3	342	75.1	237	91.9
Rs4	743	119.3	311	108.3
Rs5	795	123.6	406	122.1
Rs6	779	118.7	456	115.1
Rs7	549	76.0	227	93.3
Rs8	496	90.4	243	80.2
Rs9	501	95.3	248	93.6
Total	5,570	867.2	2,680	895.3

a The nine LGs were named Rs1 through Rs9 according to the nomenclature proposed by Shirasawa and Kitashiba.^6^

b An F_2_ mapping populations (*n* = 115) derived from a cross between an inbred line via self-pollination of radish cultivar ‘Shogoin Daikon’ and a line of *R. sativus* var. *raphanistroides* ‘Nohara 1’.

c Another F_2_ population (*n* = 95) derived from a cross between ‘Aokubi *S-h*’ and ‘Sayatori 26704’ as reported in Kitashiba et al.^2^

**Table 3:** Statistics of the ‘Okute-Sakurajima’ pseudomolecule sequences, RSAskr_r1.0.pmol

Chr^a^	#Contigs	(%)	Contig size (bp)	(%)	#Genes	(%)
Rs1	25	1.7	27,719,058	5.5	5,426	6.0
Rs2	40	2.8	42,944,316	8.5	8,295	9.2
Rs3	30	2.1	31,410,669	6.2	5,923	6.6
Rs4	35	2.4	56,498,296	11.2	10,843	12.1
Rs5	34	2.4	42,357,306	8.4	8,477	9.4
Rs6	41	2.9	53,940,652	10.7	10,403	11.6
Rs7	17	1.2	28,108,325	5.6	5,545	6.2
Rs8	30	2.1	28,319,830	5.6	5,474	6.1
Rs9	41	2.9	38,520,653	7.6	7,143	7.9
**Rs1–Rs9**	**293**	**20.4**	**349,819,105**	**69.3**	**67,529**	**75.1**
Rs0	1,144	79.6	154,715,059	30.7	22,386	24.9
Total	1,437	100.0	504,534,164	100.0	89,915	100.0

a The nine pseudomolecule sequences were named Rs1 through Rs9 according to the nomenclature proposed by Shirasawa and Kitashiba.^6^ The remaining unassigned sequences were grouped into Rs0.

Bold indicates the subtotal values of Rs1 to Rs9.

### 3.3. Genes and repetitive sequences in the ‘Okute-Sakurajima’ genome

**Figure 3:**
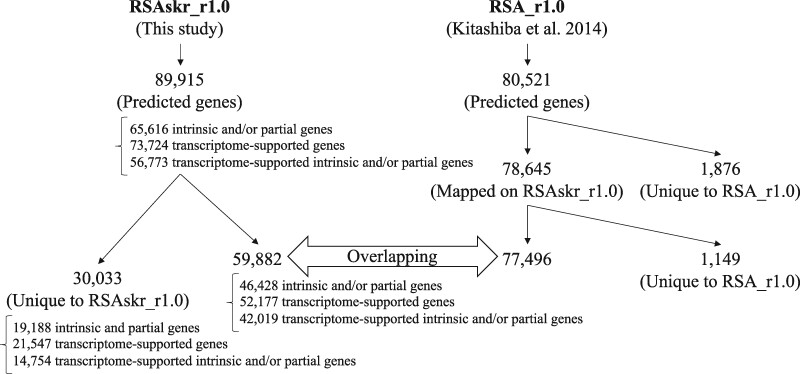
Numbers of genes predicted with the same method from the two radish genome sequences, RSAskr_r1.0 and RSA_r1.0.

We predicted a total of 89,915 likely genes including transposable elements, pseudogenes, and short genes in the current assembly RSAskr_r1.0.pmol using an *ab initio* gene prediction (Table 3 and Fig. 3), which is the same method in our previous study.^2^ Out of them, 65,616 genes were found to be intrinsic and/or partial genes and 73,724 were supported by transcriptome data that complete BUSCO score was 97.0%. In total, 56,773 genes were intrinsic and/or partial genes supported by transcriptome data. In parallel, 80,521 genes predicted in our previous assembly, RSA_r1.0,^2^ were aligned to RSAskr_r1.0.pmol to find 78,645 genes aligned on the pseudomolecules. As a result of the prediction and mapping analyses, genomic locations of 59,882 (including 42,019 transcriptome-supported intrinsic and/or partial genes) of the 89,915 predicted genes in RSAskr_r1.0 were found to overlap 77,496 of the 78,645 individual mapped genes in RSA_r1.0. The remaining 30,033 genes (=89,915 − 59,882) including 14,754 transcriptome-supported intrinsic and/or partial genes were found to exist only in the assembled sequences of ‘Okute-Sakurajima’.

**Figure 4:**
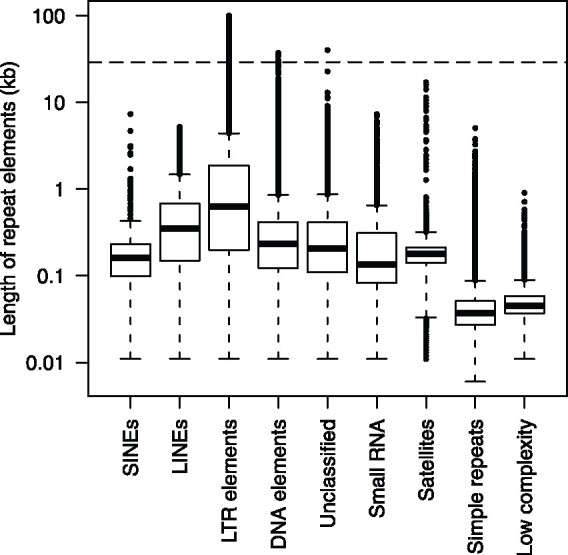
Distribution of repeat element lengths. Lengths of repeat elements were indicated by box plots. A horizontal dash line indicates the read N50 of sequence reads of ‘Okute-Sakurajima’.

**Figure 5:**
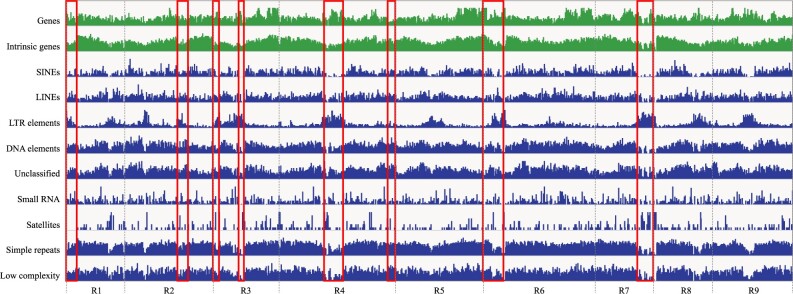
Distributions of genes and repeat elements over the ‘Okute-Sakurajima’ genome. Densities of genes and repeat elements are shown by bar plots in green and blue, respectively. Genome regions presented in only RSAskr_r1.0.pmol are represented by red boxes, which positions are corresponding to those indicated with arrows in Fig. 2.

Repetitive sequences occupied a total of 256.0 Mb (50.7%) of the RSAskr_r1.0.pmol (504.5 Mb). The repeats were consisting of nine major types with the different proportion (Table 4). Although the maximum lengths of each type were ranging from 0.9 to 100 kb (Fig. 4), the lengths of most elements were shorter than the read N50 of 29.1 kb. The genome distribution patterns were different depending on the types (Fig. 5). Out of them, the pattern of LTRs showed opposite to that of the intrinsic genes. Interestingly, the eight regions lacked in the previous study were enriched with LTRs but possessed genes as well.

**Table 4:** Repetitive sequences in the ‘Okute-Sakurajima’ sequences, RSAskr_r1.0

Repeat type	Number of elements	Length occupied (bp)	%^a^
SINEs	6,031	1,113,643	0.2
LINEs	18,132	12,792,260	2.5
LTR elements	67,265	144,417,931	28.6
DNA elements	90,424	37,170,618	7.4
Unclassified	127,034	46,043,450	9.1
Small RNA	7,177	8,492,545	1.7
Satellites	2,506	561,848	0.1
Simple repeats	112,754	5,427,620	1.1
Low complexity	25,293	1,305,261	0.3

a Percentage of sequence length of RSAskr_r1.0 (504,676,864 bp).

## 4. Conclusion and future perspective

In this study, we analysed the genome sequence of a radish cultivar named ‘Okute-Sakurajima’, a variety of ‘Sakurajima-diakon’, based on the long-read sequence technology. Advantages in this technology provided benefits to the radish genome assembly. The total assembly size of 504.5 Mb, which was roughly equal to the estimated size of 498.5 Mb, is the longest reported to date^2–5^ for any radish genome, suggesting that the long reads with the N50 length of 29.1 kb have obviously spanned the regions containing repetitive sequences in the radish genome (Fig. 4) and thus their presence did not hinder sequence assembly. The assembly contiguity represented by contig N50 in this study was remarkably longer than those in previous studies.^2–5^ Complete BUSCO score reached as high as 94.1%, among which 23.1% was duplicated BUSCOs. This was probably due to the evolutionary history of the genome triplication in this mesopolyploid species.^7^

Map-based pseudomolecules were produced which comprised 69.3% of the assembled sequences. Unexpectedly, however, we were unable to assign 30.7% of the sequences into pseudomolecules. This was largely because sequences without any SNPs are in general difficult to assemble as our genetic map construction relied on the presence of SNPs. To overcome this limitation, optical mapping and Hi-C technologies, both of which are based on physical mapping, have been developed.^9^ These technologies, when combined with traditional genetic mapping, would allow an improved genome coverage of the assembly into pseudomolecules.

In conclusion, through sequencing of the ‘Okute-Sakurajima’ genome, we have identified 89,915 genes, of which 56,773 (63.1%) were transcriptome-supported intrinsic and/or partial genes (Fig. 3). Among them, 30,033 genes (including 14,754 transcriptome-supported intrinsic and/or partial genes, 49.1%) were newly found in this study, while 59,882 genes (42,019 transcriptome-supported intrinsic and/or partial genes, 70.2%) were presented in the previous assembly.^2^ The genomic information thus expanded is expected to provide new insights into radish growth and development. Further detailed analysis in comparison with other radish cultivars will perhaps contribute to the establishment of the relevant genetic mechanisms for giant root formation as well as other useful characteristics of radish cultivars.

## Supplementary Material

dsaa010_Supplementary_Data
